# A retrospective study of neoadjuvant novel hormonal therapy prior to radical prostatectomy for high-risk prostate cancer

**DOI:** 10.3389/fonc.2025.1480861

**Published:** 2025-02-19

**Authors:** Hui Shuai, Wei Zhang, Jianping Liu, Peng Zhang, Congwang Chang, Guanghua Fu, Tao Wu

**Affiliations:** ^1^ Department of Urology, The First People's Hospital of Yibin, Yibin, Sichuan, China; ^2^ Department of Urology, Affiliated Hospital of North Sichuan Medical College, Nanchong, Sichuan, China

**Keywords:** neoadjuvant therapy, radical prostatectomy, high-risk prostate cancer, biochemical recurrence, retrospective study

## Abstract

**Purpose:**

This study aims to retrospectively describe the perioperative outcomes and short-term oncological outcomes of high-risk prostate cancer patients treated with neoadjuvant novel hormonal therapy (NNHT) combined with radical prostatectomy (RP) or RP alone.

**Materials and Methods:**

Fifty-five male patients underwent RP and were categorized based on whether NNHT was administered preoperatively. Clinical baseline characteristics, perioperative outcomes, and biochemical recurrence (BCR) rate were summarized using mean, standard deviation, medians, interquartile ranges, and frequencies. Group 1 (n=20) received NNHT in combination with RP, while Group 2 (n=35) received RP alone. Patients in the NNHT group received androgen deprivation therapy (ADT) combined with either abiraterone (1,000 mg/d), enzalutamide (160 mg/d), or apalutamide (240 mg/d) before RP. SPSS Statistics 27 was used for statistical analysis.

**Results:**

Among the 55 patients included in the study, the age, clinical T stage, N stage, biopsy Gleason scores, and the number of biopsy-positive needles appeared comparable across the two groups. However, patients in the NNHT+RP group had higher median preoperative serum prostate-specific antigen (PSA) levels (39.3 ng/mL, interquartile range [IQR]: 13.9-92.3) compared to the RP-only group (15.6 ng/mL, IQR: 10.7-19.8). The NNHT+RP group showed a lower proportion of positive surgical margins (PSM) (20%) compared to the RP-only group (49%). Similarly, the proportion of patients experiencing biochemical recurrence (BCR) within the follow-up period appeared lower in the NNHT+RP group (30%) compared to the RP-only group (57%). Additionally, operative time, hemoglobin decrease, transfusion rate, catheterization time, pathological T stage, and overall complication rates showed similar distributions across the two groups.

**Conclusion:**

This study suggests that NNHT+RP may be associated with lower rates of PSM and BCR compared to RP alone. However, further studies with larger cohorts and longer follow-up are needed to assess its long-term impact on survival and other outcomes.

## Introduction

Prostate cancer (PCa) ranks second in global male malignancies, trailing only lung cancer, and stands as the fifth leading cause of cancer-related mortality among men. In 2020, PCa accounted for 14.1% of total cancer cases (1,414,259 cases) and 6.8% of male cancer-related deaths (375,304 cases) (1, 2). China exhibits a relatively high incidence of high-risk localized and locally advanced PCa (3). The proportion of PCa patients in China who were diagnosed with intermediate to high-risk PCa at their initial diagnosis is significantly higher than that in other countries, reaching as high as 20% to 35% (4). However, treating such patients surgically poses significant challenges with limited benefits. Patients with intermediate to high-risk PCa who underwent radical prostatectomy (RP) experienced a relatively high rate of tumor recurrence. Approximately 20% of these patients exhibited biochemical recurrence (BCR) within one year post-surgery. For those with very-high-risk PCa, the BCR rate can be as high as 50% within three years following surgery (5). Consequently, researchers and clinical practitioners actively seek more effective treatment strategies to improve survival rates and the quality of life for high-risk PCa patients.

The debate over the best treatment approach for high-risk localized or locally advanced PCa is ongoing. Over the past two decades, research into neoadjuvant hormone therapy (NHT) for PCa has flourished. Some studies suggest that patients who receive NHT in conjunction with RP experience postoperative reductions in tumor staging, lower rates of positive surgical margins (PSM), reduced instances of seminal vesicle invasion, and lymph node involvement (6–8). However, other studies have failed to confirm significant benefits of NHT + RP in terms of improving biochemical recurrence-free survival, cancer-specific survival, and overall survival (9). Zhang et al. (10) conducted a meta-analysis incorporating 22 clinical studies on PCa NHT. The results showed that NHT+RP significantly reduced the rates of PSM and BCR compared to direct RP. However, these studies primarily included patients with low- and intermediate-risk PCa and lacked long-term follow-up (10). Additionally, nearly all of these trials utilized conventional hormone therapies (gonadotropin-releasing hormone agonists/antagonists or bicalutamide), 5-alpha reductase inhibitors (finasteride), or estrogenic agents as neoadjuvant treatments. In recent years, the introduction of novel hormonal agents such as abiraterone, enzalutamide, and apalutamide has generated substantial interest in high-risk PCa treatment. In the ARNEO study, Devos et al. (11) compared the efficacy of degarelix in combination with apalutamide versus degarelix monotherapy as neoadjuvant treatment before RP. Some scholars contend that the study’s control group employed androgen deprivation therapy (ADT) rather than the current standard treatment approach (no neoadjuvant treatment, direct RP). Thus, more compelling evidence from evidence-based medicine is required to challenge the prevailing standard treatment for localized PCa.

In this study, we retrospectively described the efficacy and safety of neoadjuvant novel hormonal therapy (NNHT) + RP and RP alone in the treatment of high-risk PCa. This may help in developing more effective treatment strategies for patients with high-risk PCa.

## Method

### Study design and patients

The study was conducted in accordance with the Helsinki Declaration and received approval from both our institution and the ethics committee. This retrospective single-center study was based on the STROCSS 2019 Guideline: Strengthening the reporting of cohort studies in surgery (12). All participants provided informed consent for their involvement in the study.

We retrospectively analyzed clinical data from 55 high-risk PCa patients who underwent robotic-assisted RP at the affiliated hospital of North Sichuan Medical College from March 2021 to September 2023. High risk was defined as having any one of the following: clinical stage T3, baseline PSA >20 ng/ml, or Gleason score of 8–10. All patients underwent transrectal ultrasound-guided prostate biopsy, and high-risk PCa was confirmed through pathological examination, serum prostate-specific antigen (PSA), or magnetic resonance imaging (MRI). Patients were divided into two groups: the NNHT + RP group (20 cases) and the RP group (35 cases), based on whether NNHT was administered preoperatively.

### Treatments

Patients in the NNHT group received preoperative treatment with abiraterone acetate (1000 mg/day) plus prednisone (5 mg/day), enzalutamide (160 mg/day), or apalutamide (240 mg/day) for an average duration of 4.7 months (range: 3-6 months), along with concurrent ADT via subcutaneous goserelin injections. Serum PSA levels were measured monthly. All RPs were performed by a highly experienced urologic surgeon.

### Data acquisition

We evaluated patients’ age, clinical TNM stage, biopsy Gleason score (GS), serum PSA values at diagnosis, number of biopsy positive needles, perioperative parameters, and oncological outcomes. Perioperative parameters included operation time, hemoglobin (Hb) decrease, transfusion rate, and catheterization time. Oncologic outcomes included pathological T stage, pathological GS and PSM.

Regarding postoperative follow-up assessment, we initially measured serum PSA levels at 1 month after surgery, and subsequently, we conducted routine measurements approximately every 3 months. BCR is defined as a serum PSA level equal to or exceeding 0.2 ng/ml. For patients with BCR, prostate MRI or PET/CT scanning is recommended, followed by salvage radiotherapy or adjuvant ADT as appropriate. During follow-up, patients were continuously monitored for long-term surgical complications, such as urinary incontinence, with the duration and resolution of these complications being recorded.

### Statistical analysis

Statistical analyses were performed using SPSS Statistics 27. For continuous variables, data following a normal distribution were presented as mean ± standard deviation (SD), while non-normally distributed data were summarized as median (interquartile range, IQR). The Shapiro-Wilk test was used to assess normality. Categorical variables were presented as frequencies and percentages. No statistical comparisons were conducted due to the limited sample size and baseline imbalance.

## Results

### Patients’ characteristics

Patients were divided into two groups based on whether NNHT was administered preoperatively. Group 1 (n=20) received NNHT in combination with RP, while Group 2 (n=35) received RP alone. **Table 1** provides an overview of the patients and details of our study population. The median age was 68.5 years (IQR: 60–73) in the NNHT+RP group and 71 years (IQR: 66–74) in the RP-only group. Clinical T stage, clinical N stage, biopsy Gleason score, and the number of biopsy-positive needles were similar between the two groups. However, patients in the NNHT+RP group exhibited higher preoperative serum PSA levels, with a median PSA of 39.3 ng/mL (IQR: 13.9–92.3) compared to 15.6 ng/mL (IQR: 10.7–19.8) in the RP-only group. All patients undergoing NNHT showed good tolerance to novel hormonal agents and goserelin acetate. Only one patient experienced a rash and mild bilateral lower limb edema. No patients presented with severe cardiac disease, hepatic or renal toxicity, fractures, gastrointestinal reactions, or neurological involvement.

**Table 1:** Baseline clinicopathological characteristics.

	Neoadjuvant novel hormonal therapy + RP	RP
No. of patients	20	35
Median age, years median (IQR)	68.5 (60-73)	71 (66-74)
Clinical T stage at diagnosis		
T2	8 (40%)	21 (60%)
T3a	4 (20%)	5 (14%)
T3b	8 (40%)	9 (26%)
Clinical N stage at diagnosis		
N1	3 (15%)	3 (9%)
Biopsy Gleason score		
7	4 (20%)	8 (23%)
8	6 (30%)	16 (46%)
9-10	10 (50%)	11 (31%)
Median PSA at diagnosis, ng/mL median (IQR)	39.3 (13.9-92.3)	15.6 (10.7–19.8)
<10	2 (10%)	6 (17%)
10-20	6 (30%)	22 (63%)
>20	12 (60%)	13 (37%)
Number of biopsy positive needles, mean ± SD	7.4 ± 1.9	6.3 ± 2.2

RP, radical prostatectomy; IQR, interquartile range; SD, standard deviation.

### Perioperative, pathological, and oncologic outcomes

Postoperative parameters for both groups are summarized in **Table 2**. The median operative time was 220.8 minutes (IQR: 177.8–266.1) in the NNHT+RP group and 228.0 minutes (IQR: 202.2–285.0) in the RP-only group. Hb decrease and transfusion rates were comparable between groups. The mean catheterization times were 15.2 ± 2.1 days and 14.9 ± 2.0 days in the NNHT+RP and RP-only groups, respectively. Pathological T stage distributions showed a higher proportion of complete pathological responses (pCR) in the NNHT+RP group (25%) compared to the RP-only group (0%). Seminal vesicle invasion was observed in 10% of patients in the NNHT+RP group and 31% in the RP-only group. Pathological Gleason score distributions were similar between groups, with the majority of patients in both groups having scores of 9–10. The PSM rate was less frequent in the NNHT+RP group (20%) compared to the RP-only group (49%). Similarly, the proportion of patients experiencing BCR within the follow-up period appeared lower in the NNHT+RP group (30%) compared to the RP-only group (57%).

**Table 2:** Postoperative parameters.

	Neoadjuvant novel hormonal therapy+RP (*n*=20)	RP (*n*=35)
Operative time (min), media (IQR)	220.8 (177.8-266.1)	228.0 (202.2-285.0)
Hemoglobin decrease (g/L)	14.5 (10.0-23.3)	18.0 (14.0-23.0)
Transfusions	2 (10%)	3 (9%)
Catheterization time (d), mean ± SD	15.2 ± 2.1	14.9 ± 2.0
Pathological T stage		
pT2	9 (45%)	9 (26%)
pT3a	4 (20%)	15 (43%)
pT3b	2 (10%)	11 (31%)
pCR	5 (25%)	0 (0%)
Seminal vesical invasion	2 (10%)	11 (31%)
Pathological Gleason score		
7	1 (5%)	6 (17%)
8	4 (20%)	13 (37%)
9-10	10 (50%)	16 (46%)
pCR	5 (25%)	0 (0%)
Positive surgical margin	4 (20%)	17 (49%)
Biochemical recurrence	6 (30%)	20 (57%)

RP, radical prostatectomy; IQR, interquartile range; SD, standard deviation; pCR, pathological complete response.

### Surgical complications

Regarding intraoperative and postoperative adverse events (**Table 3**), both groups of patients experienced various complications, including rectal injury, lung infection, early and continuous urinary incontinence, urethral stricture, lymphorrhea, and venous thrombosis. In this study, there were no patient fatalities, and no complications classified as Clavien–Dindo grade III or higher occurred. The incidence of complications was similar between the two groups.

**Table 3:** Surgical complications.

	NNHT+RP (*n*=20)	RP (*n*=35)
Rectal injury	1	1
Conversion to open surgery	0	0
Transfusions	1	3
Lung infection	3	5
Early urinary incontinence	12	21
Continuous incontinence	0	1
Urethral stricture	1	0
Lymphorrhea	2	2
Venous thrombosis	1	2

## Discussion

For patients with localized PCa, RP and radical radiotherapy are generally considered the preferred treatment strategies (13). RP holds a dominant position in the treatment of localized PCa (14). However, high-risk PCa patients faced a high risk of BCR and disease progression after local treatment. Approximately two-thirds of prostate cancer-specific mortality in men with localized disease is attributed to high-risk or very high-risk groups (15). This unfavorable prognosis may, in part, be attributed to tiny metastatic lesions that conventional imaging cannot capture at the time of diagnosis (16). Preoperative neoadjuvant endocrine therapy was considered to have a positive impact on improving patient outcomes. It aided in reducing tumor volume, lowering staging, and eliminating those tiny metastatic lesions that were difficult to detect with routine imaging (17). However, because previous studies suggested that NHT alone did not significantly improve overall survival (9), the guidelines did not recommend neoadjuvant therapy as a standard treatment option (18). Recent years may see an improvement in the situation with the introduction of novel androgen receptor inhibitors. Furthermore, while guidelines typically do not endorse NHT for PCa patients, there exists a subset of individuals with high-risk localized or locally advanced PCa who face challenges such as tumors that are too large for surgical resection, invasive growth, or high surgical risks that preclude surgical intervention. In such scenarios, NHT may hold the potential to reduce cancer staging, diminish tumor volume, or alleviate the complexity of surgery (17), thereby affording patients an opportunity for surgery and potentially extending their survival. Therefore, the potential benefits of neoadjuvant therapy should not be dismissed outright.

In China, the incidence of PCa has been steadily increasing year by year due to the escalating aging population and the widespread promotion of PCa screening (19). As a result, there is a growing number of high-risk PCa patients (3). Chinese medical research institutions and hospitals actively engage in PCa research and clinical trials, aiming to continuously enhance diagnostic and treatment methods while relentlessly seeking innovative therapeutic strategies. We conducted a retrospective study to compare the efficacy of NNHT + RP and RP alone in the treatment of high-risk PCa. Current evidence shows that NNHT + RP group offers favorable oncologic outcomes compared with RP only group, including lower PSM rate and longer BCR-free survival.

The effect of neoadjuvant therapy on RP remains controversial. According to a previously published research, hormone therapy contributed to the reduction of hemorrhage and the alleviation of surgical complexity (20). However, Polito (21) and Soloway et al. (22) reported that the prostate following neoadjuvant therapy exhibited inflammation infiltration, stromal fibrosis, and seminal vesicle adhesions. This perspective suggested that NHT prior to RP may render the surgery more challenging. In Soloway et al.’s study (22), although a higher percentage (7% versus 5%) of challenging dissections can be seen in patients treated with androgen deprivation, there was no significant difference in either the operative time (243.8 versus 234.4 minutes) or the extent of intraoperative bleeding (694.4 versus 656.7 ml) between the two groups. Our study results showed that operative time, Hb decrease, transfusion rate, and catheterization time were comparable between groups. To further determine the effect of neoadjuvant therapy before RP, prospective multicenter large-cohort studies are needed.

It is noteworthy that many healthcare centers are currently adopting robot-assisted systems for RP (23). With the assistance of flexible robotic arms and 3-dimensional vision, surgeons can perform complex laparoscopic surgeries with greater precision and effectiveness. The advanced capabilities of robotic surgical systems, surpassing traditional surgical visualization and unparalleled precision, may potentially mask the increased anatomical complexities resulting from neoadjuvant therapy. As a result, it is imperative to exercise caution when making comparisons with previous studies evaluating the efficacy of NHT prior to laparoscopic RP.

BCR is a widely recognized intermediate endpoint for localized PCa, commonly used in clinical practice to guide salvage therapy. Hu et al. (24) evaluated 48 patients with intermediate- or high-risk PCa who were treated with NHT varied from 2 months to 12 months and those with non-NHT before robot-assisted RP. The PSM and BCR rate were significantly lower in the NHT group. Ravi et al. (25) conducted a comparative analysis to compare outcomes between neoadjuvant therapy with a novel hormonal agent prior to RP and RP alone in patients with high-risk PCa. The study concluded that NNHT prior to RP was associated with longer time to BCR and superior metastasis free survival (MFS) compared to RP alone in men with high-risk PCa. These findings are currently being studied in the phase 3 PROTEUS trial (NCT03767244). In our study, the NNHT+RP group exhibited lower rates of PSM and BCR. Moreover, pCR was observed in five patients following NNHT.

The duration of neoadjuvant therapy may potentially impact BCR following RP. Presently, due to a lack of robust evidence, a consensus has not been reached regarding the optimal duration of NHT. A prospective phase III clinical trial suggested that biochemical and pathological regression of prostate tumors continued to occur between 3 and 8 months of preoperative hormonal therapy, indicating that the optimal duration of this treatment might exceed 3 months (26). In Pu et al.’s study (27), there was no significant difference in PSM and BCR rates between patients receiving 3 months and 8 months of NHT. However, a Cochrane systematic review and meta-analysis concluded that prolonged NHT can reduce the incidence of PSM (OR=0.56, 95% CI 0.39-0.80, *p* = 0.002) following RP (28). Additionally, Hu et al. (24) evaluated the efficacy of combining NHT with robot-assisted RP in patients with intermediate- to high-risk PCa. Their findings demonstrated that patients receiving 4-12 months of NHT had a significantly lower BCR rate compared to those who received NHT for 2-3 months (*p* = 0.0133). In most current studies on NHT for PCa, the duration of preoperative neoadjuvant treatment is generally between 3 to 6 months. Therefore, prospective multicenter large-scale cohort studies are still needed to further elucidate the optimal duration of preoperative hormone therapy.

Comparing intraoperative and postoperative complications between the two groups, we found no statistically significant differences in any of the complications. Clavien-Dindo grade 3 or higher complications were rare in both groups and are recognized risks associated with RP. Most complications were promptly identified and appropriately managed. Urinary incontinence is the most common complication following RP, with the vast majority of patients experiencing varying degrees of incontinence after catheter removal. The impact of urethral reconstruction techniques on incontinence remains a subject of debate. Whether one reconstruction technique is superior to others requires further investigation. Complete preservation of the neurovascular bundles is currently considered an effective perioperative intervention for improving early urinary incontinence.

This study has several limitations. Firstly, it is a retrospective design with a relatively small sample size in both comparison groups, which limits the statistical power and generalizability of the findings. Additionally, the follow-up duration was limited, and a small number of patients were lost to follow-up, further restricting the ability to draw conclusions regarding long-term outcomes. Secondly, selection bias may have influenced the results, as patients undergoing NNHT + RP had relatively higher preoperative PSA levels. Higher PSA levels likely influenced the decision to opt for neoadjuvant therapy, which could introduce bias in treatment allocation. As a result, the observed outcomes may not fully reflect the broader high-risk prostate cancer population. Moreover, this study was unable to perform subgroup analyses based on individual NNHT agents. Due to national insurance policies and economic constraints in China, most patients in our cohort received abiraterone as their neoadjuvant therapy. Given these limitations, there is a need for larger-scale, prospective studies with longer follow-up periods to comprehensively evaluate the clinical efficacy and long-term outcomes of NNHT prior to RP in high-risk prostate cancer patients.

## Conclusion

In this preliminary study, NNHT prior to RP in high-risk PCa patients appeared to reduce rates of PSM and BCR without increasing surgical complexity, operative time, or blood loss. Further studies with longer follow-up and larger cohorts are needed to evaluate its impact on survival outcomes.

## Data Availability

The original contributions presented in the study are included in the article/supplementary material. Further inquiries can be directed to the corresponding author.
